# Repetitive transcranial magnetic stimulation may promote the reversion of mild cognitive impairment to normal cognition

**DOI:** 10.3389/fpsyt.2025.1544728

**Published:** 2025-04-03

**Authors:** Zhiwei Guo, Yi Jiang, Jiayuan He, Ning Jiang

**Affiliations:** ^1^ National Clinical Research Center for Geriatrics, West China Biomedical Big Data Center, West China Hospital, Sichuan University, Chengdu, China; ^2^ Institute of Rehabilitation and Imaging of Brain Function, The Second Clinical Medical College of North Sichuan Medical College, Nanchong Central Hospital, Nanchong, China

**Keywords:** Alzheimer’s disease, functional connectivity, cognitive reversion, mild cognitive impairment, resting state network

## Abstract

**Purpose:**

This study aimed to investigate the potential effects of repetitive transcranial magnetic stimulation (rTMS) on the reversion of mild cognitive impairment (MCI) to normal cognitive function and to elucidate the underlying mechanisms.

**Methods:**

The study enrolled 25 MCI participants, who underwent a 10-day of rTMS treatment and an 18-month follow-up, along with 15 healthy subjects. Participants with MCI were categorized into MCI reverters (MCI-R) and MCI maintainers (MCI-M). We assessed differences in baseline cognitive performance, functional connectivity, and changes of cognitive functions after rTMS between MCI-R and MCI-M to identify possible predictors of reversion of MCI and explore the neural modulation mechanisms.

**Results:**

MCI-M exhibited more severe cognitive impairments across more domains, particularly in language function (p < 0.05). Functional connectivity was more severely damaged in MCI-M participants, notably within the default mode network (DMN), executive control network (ECN), and frontoparietal network (FPN). After rTMS therapy, MCI-R participants demonstrated more significantly improved immediate and delayed recall memory scores (p < 0.05). These memory function changes and baseline functional connectivity of DMN, ECN, and FPN were predictive of the reversion of MCI.

**Conclusions:**

The efficacy of rTMS in memory function may promote the reversion of MCI to normal cognition, with the functional connectivity of DMN, ECN, and FPN playing a crucial important role. The severity of cognitive impairment and functional connectivity damage correlated with the likelihood of the reversion of MCI to normal cognition, underscoring the importance of early rTMS intervention for dementia prevention.

## Introduction

Mild cognitive impairment (MCI) is an intermediate stage between normal cognitive aging and dementia (1, 2), characterized by a gradual progressive cognitive decline. MCI is considered a clinical precursor of Alzheimer’s disease (AD) and is associated with an elevated risk of dementia. However, not all individuals with MCI progress to dementia, some may revert to normal or maintain stability (3, 4). Early intervention, such as cognitive training, and the absence of affective symptoms are believed to be associated with reversion and are considered modifiable factors (4, 5). Current research has paid less attention to early treatments aimed at reversing MCI to normal cognitive function, and the mechanisms involved remain unclear. Therefore, investigating the effects and neural mechanisms of early intervention in MCI patients who revert to normal cognitive function could aid in developing specific early intervention strategies and is crucial for the early prevention of dementia.

Given the limited efficacy of clinical pharmacological interventions in treating AD and MCI, nonpharmacological treatments in MCI have garnered more attention in recent years. Non-invasive brain stimulation techniques, such as repetitive transcranial magnetic stimulation (rTMS) has been considered potential effective nonpharmacological treatment methods for various neuropsychiatric diseases. Previous studies have demonstrated the positive effects of rTMS on improving cognitive functions of MCI patients, including global cognitive function, memory function, language function, and executive function (6–9). Both short-term and long-lasting therapeutic effects of rTMS on MCI have been reported (6–9). Additionally, brain imaging studies suggest that rTMS could modulate spontaneous brain activity (10, 11), structural and functional connectivity (12–14) in cognitive-related brain areas of MCI and AD. These functional connectivity changes post-rTMS therapy have shown significant correlation with cognitive improvement and could serve as a valuable imaging markers to predict the after effects of rTMS (15–17). However, the potential effects of rTMS on the reversion of MCI to normal and the underlying mechanisms have not been reported.

Numerous neuroimaging meta-analyses have demonstrated aberrant regional brain activity, functional connectivity, cerebral blood flow, and structural changes in MCI and AD patients relative to healthy elderly people (18–22). The locations of these consistent abnormal changes are predominantly associated with cognition-related brain networks involving the default mode network (DMN), salience network (SN), and executive control network (ECN), and frontoparietal network (FPN) (19, 20, 23, 24). Besides, the functional connectivity of these networks could be regulated by rTMS (25, 26), transcranial direct current stimulation (tDCS) (27), physical exercise (28, 29), and acupuncture (30). Therefore, the cognitive-related resting-state networks (RSNs) may be able to stably reflect the dynamic modulation of rTMS in brain functional connectivity in MCI patients, thereby achieving the goal of assessing the prognosis and efficacy of rTMS treatment.

The objective of our study was to determine the effects of rTMS on reversion of MCI to normal cognitive function and to investigate the underlying mechanisms. We identified MCI individuals who reverted to normal cognitive function by assessing cognitive performance at 18 months post-rTMS. Comparison of baseline cognitive performance, baseline functional connectivity, and changes of cognitive function after rTMS between MCI participants who revert to normal and who not were conducted to explore possible factors that influencing the reversion. Additionally, we assessed the relationships between changes in cognitive function and changes in functional connectivity with the reversion of MCI to clarify the impact and underlying neural modulation mechanisms of rTMS efficacy on the reversion of MCI. We hypothesized that rTMS therapy would promote the reversion of MCI to normal, with improvements in cognitive performance and baseline functional connectivity predicting the reversion.

## Materials and methods

### Participants

Participants were recruited from the local community through advertisements. A total of 53 MCI participants were included in our study and completed a 10-day rTMS therapy. Twenty-five of these MCI participants completed an 18-month follow-up after therapy. Fifteen healthy elderly individuals were also included as a normal control group (NC). All participants provided written informed consent after understanding the study procedure. The study was approved by the Ethics Committee of the Second Clinical Medical College of North Sichuan Medical College and the Ethics Committee on Biomedical Research, West China Hospital of Sichuan University in accordance with the Declaration of Helsinki.

According to the National Institute on Aging and the Alzheimer’s Association (NIA-AA) guidelines (31), MCI participants were enrolled if they met the following inclusion criteria: (1) aged 55–80 years; (2) had relevant symptoms of cognitive impairment reported by the patient or their family members or confirmed by the clinical physician; (3) had impairment in one or more cognitive domains confirmed by cognitive tests (scores on the Mini-Mental State Examination: illiterate > 17, elementary school > 20, junior high school and above > 24); (4) had a Clinical Dementia Rating (CDR) score of 0.5; (5) maintained general independent living abilities, with mild impairment in complex instrumental activities of daily living; and (6) test scores not meeting the diagnostic criteria for dementia. Exclusion criteria for all participants included: (1) a history of neurological/psychiatric diseases (e.g., stroke, epilepsy, Parkinson’s disease, traumatic brain injury, etc.) that may lead to cognitive decline; (2) congenital mental and cognitive retardation; (3) systematic diseases (e.g., syphilis, thyroid dysfunction, anthracemia, severe anemia, or HIV) that could cause cognitive impairment; (4) addiction or treatment that may influence cognitive ability; or (5) inability to complete neuropsychological assessments or contraindication for MRI and rTMS.

NC participants matched for age and gender were required to meet the following criteria: (1) no memory complaints; (2) a Clinical Dementia Rating (CDR) score of 0; (3) normal cognitive function.

### rTMS treatment protocol

All MCI participants received rTMS at a frequency of 10Hz once a day, 5 days per week, for a continuous two-week period. In each session, rTMS was applied over the dorsolateral prefrontal cortex (DLPFC) using a YRD CCY-II stimulator with a figure-of-eight coil (90% rest motor threshold (RMT); 50 pulses over 5s; 30 trains; inter-train interval of 25s; 1500 pulses daily; 14 mins, 35s). Before treatment, each participant first underwent the measurement of their RMT. During the measure of RMT, the participant was seated comfortably in a chair and instructed to relax. Then the TMS coil was positioned over the primary motor cortex, corresponding to the hand motor area. The TMS device is set to deliver pulse stimulation starting at a low intensity, gradually increasing the output. Finally, the RMT was defined as the minimal output of stimulation that could evoke a muscle twitch of the contralateral fist dorsal interosseous.

Cognitive performance and MRI data were assessed prior to treatment (baseline) and immediately following treatment completion. MCI participants willing to undergo an 18-month follow-up were also provided with a comprehensive cognitive assessment and an MRI examination at follow-up.

### Clinical outcome measures

Comprehensive clinical and neuropsychological assessments included: (1) general cognitive performance, namely, the Mini-Mental State Examination (MMSE) and the Huashan version of the Montreal Cognitive Assessment (MoCA); (2) memory function, namely, the Auditory Verbal Learning Test (AVLT), including immediate recall (AVLT-I), delayed recall (AVLT-DR), and recognition (AVLT-R); (3) language function, namely, the Boston Naming Test (BNT) and the Animal Semantic Fluency Test (AFT); and (4) executive function, namely, the Shape Trails Test (STT), consisting of two parts, A and B. Pre- and post- changes in these cognitive scores were calculated to evaluate the efficacy of rTMS intervention.

During the follow-up, all participants were diagnosed based on their cognitive assessment scores and the diagnostic criteria for MCI and AD. Thirteen MCI participants were diagnosed as MCI maintainers (MCI-M), the remaining MCI participants had reverted to normal cognitive function and were defined as MC reverters (MCI-R).

### MRI data acquisition

Similar to the evaluation of cognitive performance, each MCI participant underwent an MRI examination (on a Philips Ingenia CX 3.0 T scanner) at baseline, immediately after the final treatment session, and at follow-up. Individuals in the NC group underwent an MRI scan only once at the time of enrollment. High-resolution T1-weighted anatomical imaging (1 mm^3^) and resting-state functional magnetic resonance imaging (rs-fMRI) were performed. T1-weighted anatomical images were scanned along the sagittal plane with parameters as follows: repetition time/echo (TR/TE) = 6.67/3.02 ms, field of view = 240 × 240.0 mm^2^, flip angle = 8°, acquisition matrix = 240 × 240, voxel size = 1.0 × 1.0 × 1.0 mm^3^, slices = 170. During the fMRI scan, patients were instructed to stay awake, relax with their eyes closed and remain motionless as possible. The rs-fMRI images were acquired with the following parameters: TR/TE = 2,000/30 ms, field of view = 240.0 × 240.0 mm^2^, flip angle = 90°, matrix = 64 × 64, voxel size = 3.75 × 3.75 × 3.75 mm^3^, and 36 axial slices. A total of 245 volumes were continuously obtained for each scan.

### Preprocessing and group independent component analysis

Preprocessing of the rs-fMRI data was conducted by using the SPM 12 (http://www.fil.ion.ucl.ac.uk/spm) software package, which included slice timing, spatial head motion realignment, normalization, and smoothing. Prior to the preprocessing procedure, the first 5 volumes of the fMRI datasets of each patient were discarded to eliminate magnetization equilibrium effects and account for the adaptation phase of the participants. After preprocessing, patients with a maximum head translation greater than 3 mm or a maximum rotation of 2° were excluded from subsequent analysis.

After spatial preprocessing, group-level independent component analysis (ICA) was performed by using the GIFT v4.0b toolbox (https://trendscenter.org/software/gift/). Both baseline and post-rTMS datasets were used for this analysis. Two data reduction steps were conducted by using principal component analysis (PCA). The minimum description length (MDL) criterion was used to automatically estimate the number of independent components (ICs) to retain in the subsequent ICA stage. Then, the infomax algorithm (32) was applied to decompose the reduced data of all patients into 33 estimated ICs. This calculation process was repeated multiple times for estimation of the stability (33). Participant-specific time courses and spatial maps were obtained from the spatiotemporal regression back reconstruction approach (34), and the results were transformed to z scores.

Cognitive-related resting-state networks (RSNs) were selected for subsequent analysis based on previously reported RSNs in fMRI. Components were identified through visual observation. Previous studies have demonstrated that MCI is associated with widespread aberrant brain activity, alterations in functional connectivity, and structural changes, predominantly within several cognition-related brain networks, such as the DMN, SN, and ECN (18–22). Additionally, the left FPN (LFPN) and right FPN (RFPN) were reported to be related to the cognitive performance of MCI (35, 36) and showed significant correlation with the clinical efficacy of physical exercise (28) and rTMS intervention (26). Therefore, these RSNs were selected and extracted from the independent components according to their anatomical and functional properties to identify possible imaging biomarkers for MCI patients who reverted to normal after rTMS. These networks included the anterior DMN (aDMN), posterior DMN (pDMN), SN, insula network (IN), ECN, hippocampal network (HN), LFPN, and RFPN.

### Statistical analysis

Statistical analysis of RSNs was performed using the SPM 12 software package. To investigate functional connectivity differences, comparisons of baseline intra- and inter-functional connectivity of RSNs among MCI-M, MCI-R, and NC were conducted using the two-sample t-test. Paired t-tests were used to examine the changes of functional connectivity after rTMS therapy relative to baseline.

Statistical analysis of demographic information and neurocognitive scores was performed using IBM Statistical Package for Social Sciences (SPSS) v23.0. Group differences in age, years of education (1–6 years: elementary school; 7–9 years: junior secondary school; 10–12 years: senior secondary school; > 12 years: university or college or its equivalent), and cognitive assessment scores among MCI-R, MCI-M, and NC groups were examined with one-way ANOVA or nonparametric test (Kruskal-Wallis) according to the results of normality and homogeneity. *Post hoc* pairwise t-tests or nonparametric tests (Mann-Whitney) for multiple comparisons were performed if the comparison among the three groups yielded significant results (P < 0.05). The comparison of the gender information between two groups was conducted by using the chi-square test. Changes of cognitive performance after rTMS were calculated for MCI-R and MCI-M respectively. Similarly, two-sample t-tests or nonparametric tests (Mann-Whitney) were used to evaluate the differences in rTMS efficacy between MCI-R and MCI-M groups.

Based on the comparison results of functional connectivity, information of the brain regions showing significant intra- and inter- functional connectivity differences between groups and within each group were extracted. The spearman correlation coefficients between the functional connectivity of these brain regions at baseline and follow-up and the cognitive score changes after rTMS treatment were calculated. This can help us understand the relationship between baseline functional connectivity and the efficacy of rTMS, as well as the impact of rTMS efficacy on cognitive status at follow-up.

Logistic regression analysis was used to identify baseline functional connectivity, cognitive performance, and cognitive improvement scores that were significantly correlated with the reversion of MCI at follow-up. Cognitive improvement scores can reveal the efficacy of rTMS, and cognitive performance at 18 months follow-up can reflect the reversion of MCI to normal. Therefore, according to the results of regression analysis, we can evaluate the influence of rTMS efficacy and baseline functional connectivity on the reversion from MCI to normal.

Finally, receiver operating characteristic curve (ROC) analysis was conducted to evaluate the predictive value of baseline functional connectivity and cognitive improvement scores post-rTMS for the reversion of MCI. Functional connectivity in brain regions showing significant group differences was used as the index during the ROC analysis. The area under the curve (AUC), sensitivity, and specificity were calculated for each analysis.

## Results

### Demographic and baseline cognitive characteristics

**Figure 1:**
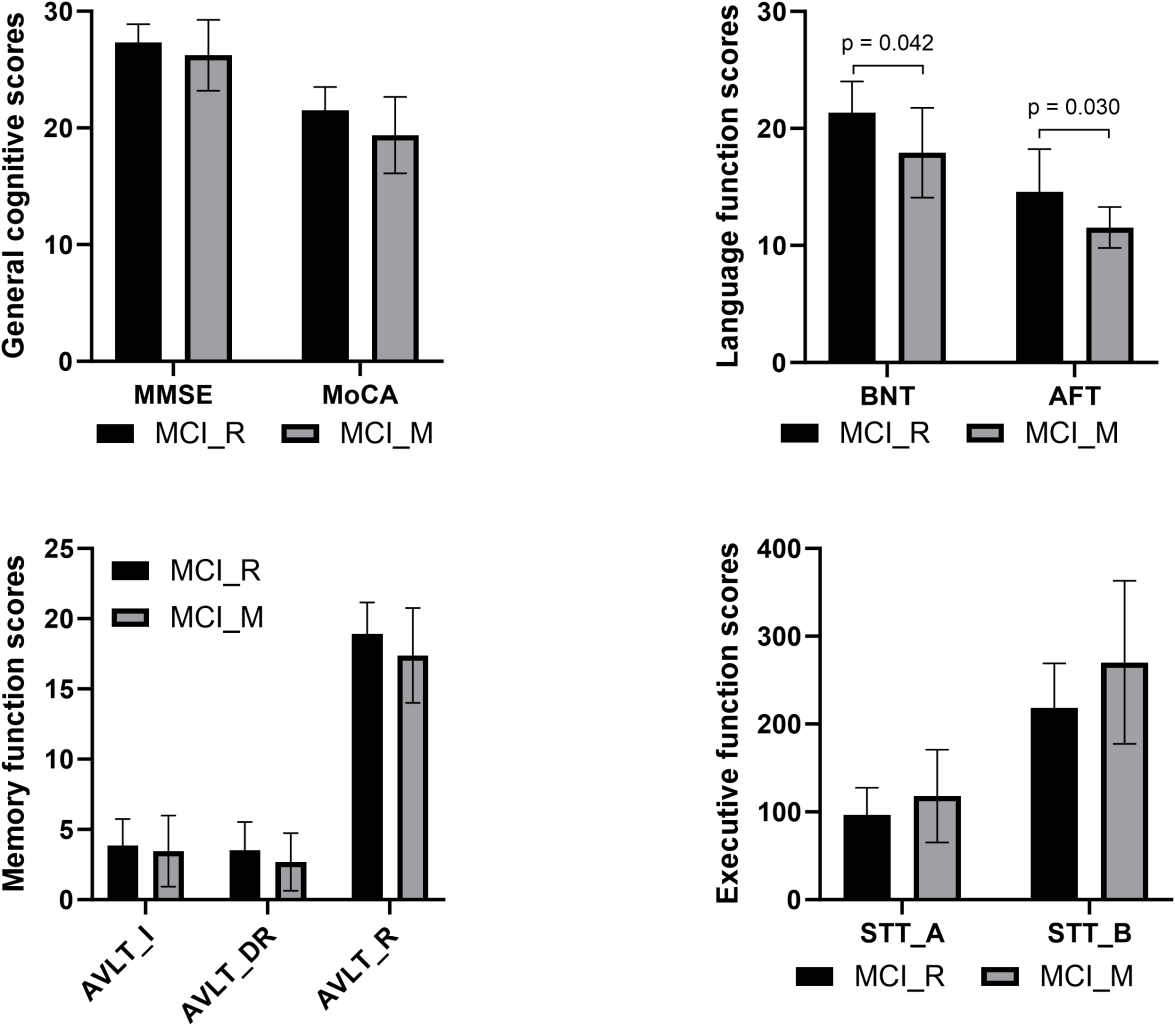
Comparison of baseline neurocognitive performance between MCI-R and MCI-M showed significant differences in BNT and AFT.

Baseline demographic information and neurocognitive performance data for participants in MCI-M, MCI-R, and NC are presented in **Table 1**. Education and the cognitive assessment scores of MoCA, AVLT-I, AVLT-DR, AVLT-R, BNT, AFT, STT-A, and STT-B showed significant differences between MCI-M and NC (p < 0.05). For MCI-R participants, age and the cognitive assessment scores of MoCA, AVLT-I, AVLT-DR, AVLT-R, STT-A, and STT-B showed significant differences relative to NC (p < 0.05). Additionally, compared to MCI-R, MCI-M showed significantly lower BNT and AFT scores (p < 0.05) (**Figure 1**). From the comparison among MCI-R, MCI-M and NC, we observed that MCI-M participants presented more severe cognitive impairment across more domains and a lower education level. Furthermore, language abilities, including naming and language fluency were more severely damaged.

**Table 1:** Characteristics and neurocognitive scores of MCI and NC participants.

	MCI-R (N = 12)	MCI-M (N = 13)	NC (N = 15)
Age	73.27 ± 4.71^$^	70.18 ± 4.42	67.33 ± 6.48
Gender (M/F)	4/8	2/11	6/9
Education	7.92 ± 2.81	7.46 ± 2.37^#^	9.73 ± 1.98
MMSE	27.33 ± 1.56	26.23 ± 3.03	28.13 ± 1.64
MoCA	21.50 ± 2.02^$^	19.38 ± 3.28^#^	24.47 ± 2.95
AVLT-I	3.83 ± 1.90^$^	3.46 ± 2.54^#^	6.40 ± 2.26
AVLT-DR	3.50 ± 2.02^$^	2.69 ± 2.06^#^	6.60 ± 1.99
AVLT-R	18.92 ± 2.23^$^	17.38 ± 3.38^#^	21.93 ± 1.62
BNT	21.33 ± 2.67	17.92 ± 3.84*^#^	22.53 ± 2.26
AFT	14.58 ± 3.65	11.54 ± 1.76*^#^	16.40 ± 4.34
STT-A	96.33 ± 31.07^$^	118.08 ± 52.88^#^	69.47 ± 18.77
STT-B	218.42 ± 50.90^$^	270.23 ± 92.82^#^	164.27 ± 35.33

^*^ Significant difference (p < 0.05) between MCI-R and MCI-M; ^$^ Significant difference (p < 0.05) between MCI-R and NC; ^#^ Significant difference (p < 0.05) between MCI-Mand NC; M, Male; F, Female; MMSE, Mini-Mental State Examination; MoCA, Montreal Cognitive Assessment; BNT, Boston Naming Test; AVLT-I, auditory verbal learning test-immediate recall; AVLT-DR, auditory verbal learning test-delayed recall; AVLT-R, auditory verbal learning test-recognition; AFT, Animal Verbal Fluency Test; STT-A, Shape Trails Test Part A; STT-B, Shape Trails Test Part B.

### Efficacy difference of rTMS in MCI-R and MCI-M

To determine whether the reversion of MCI was related to different therapeutic effects of rTMS on cognitive functions, a comparison of cognitive score changes after rTMS between MCI-R and MCI-M groups was conducted. Higher improvement was observed in AVLT-I (z = 2.614, p = 0.009) and AVLT-DR (z = 2.326, p = 0.020) after the 10-day of rTMS therapy in MCI-R participants compared with MCI-M participants. These results suggest that MCI patients who reverted to normal may have obtained more benefits from rTMS therapy, particularly in immediate and delayed recall memory functions. As for safety, the rTMS treatment was well tolerated, with no adverse events during the experimental procedure. Even those patients who received higher stimulation intensity did not complain about painful or uneasiness sensations.

### Differences of intra-functional connectivity within RSNs between-groups and changes within-group

Comparison of the neurocognitive performances detected significant differences between MCI-M and MCI-R. Theoretically, there should be consistent functional connectivity differences in RSNs. These baseline functional connectivity differences may also be related to different reversion outcome at follow-up. Therefore, we tested for differences in intra-functional connectivity of each RSN between MCI-M and MCI-R, and between MCI and NC.

**Figure 2:**
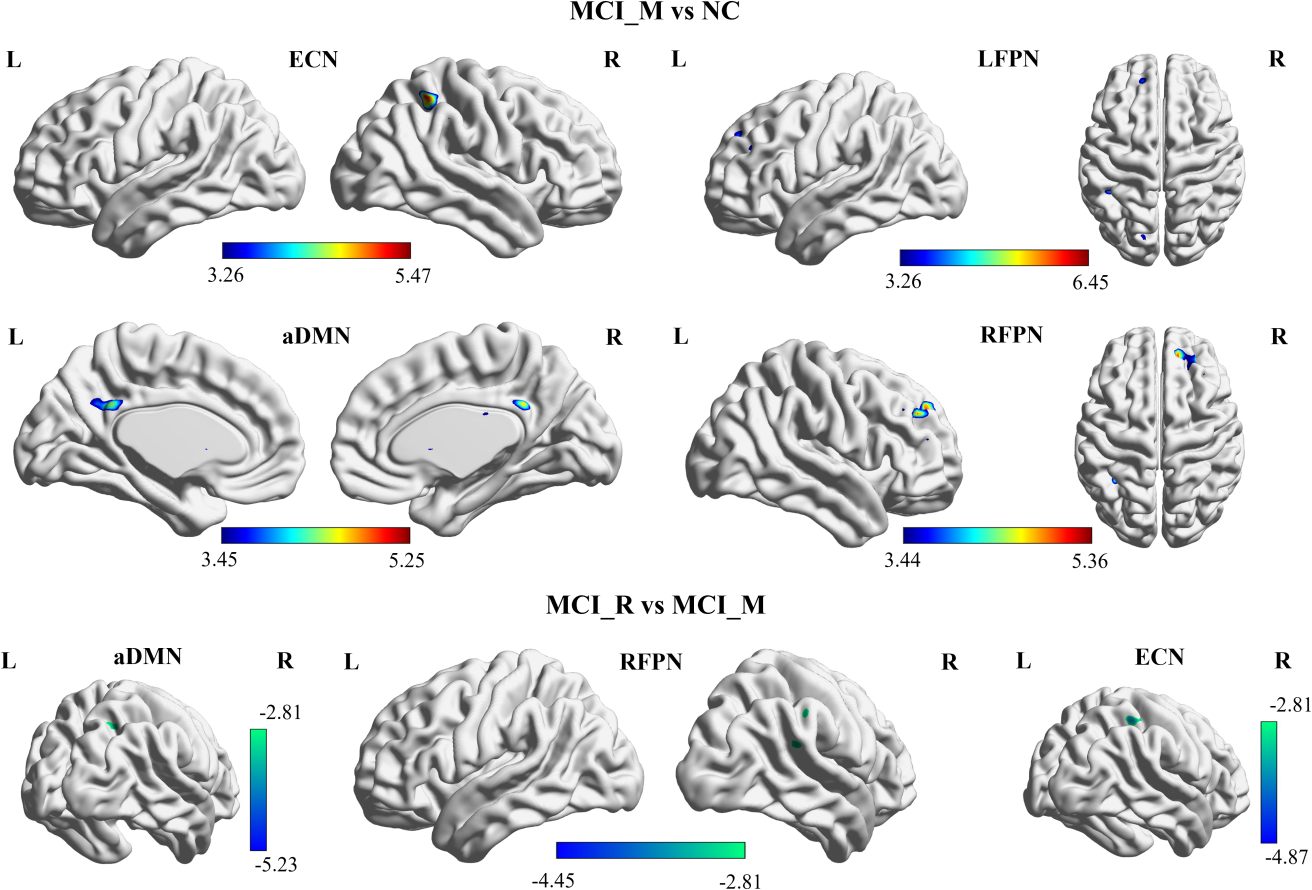
Baseline functional connectivity of the RSNs showed significant difference between MCI-M and NC in aDMN, ECN, LFPN, and RFPN (p< 0.05, FDR corrected); and showed significant difference between MCI-M and MCI-R in aDMN, ECN, and RFPN (p< 0.05, alphasim corrected). MCI-M, MCI maintainers; MCI-R, MCI reverters; L, left; R, right; aDMN, anterior default mode network; ECN, executive control network; LFPN, left frontoparietal network; RFPN, right frontoparietal network.

Compared to NC, MCI-M showed significantly increased functional connectivity in the left posterior cingulate cortex and left precuneus in pDMN; right inferior parietal lobe in ECN; left superior frontal gyrus, left middle frontal gyrus, and left inferior parietal lobe in LFPN; and in right superior frontal gyrus and right middle frontal gyrus in RFPN (p < 0.05, FDR corrected). However, no significant results were observed between MCI-R and NC. These results may suggest that the damage to the intra-functional connectivity of DMN, ECN, LFPN, and RFPN is more severe in MCI-M participants (**Figure 2**).

**Figure 3:**
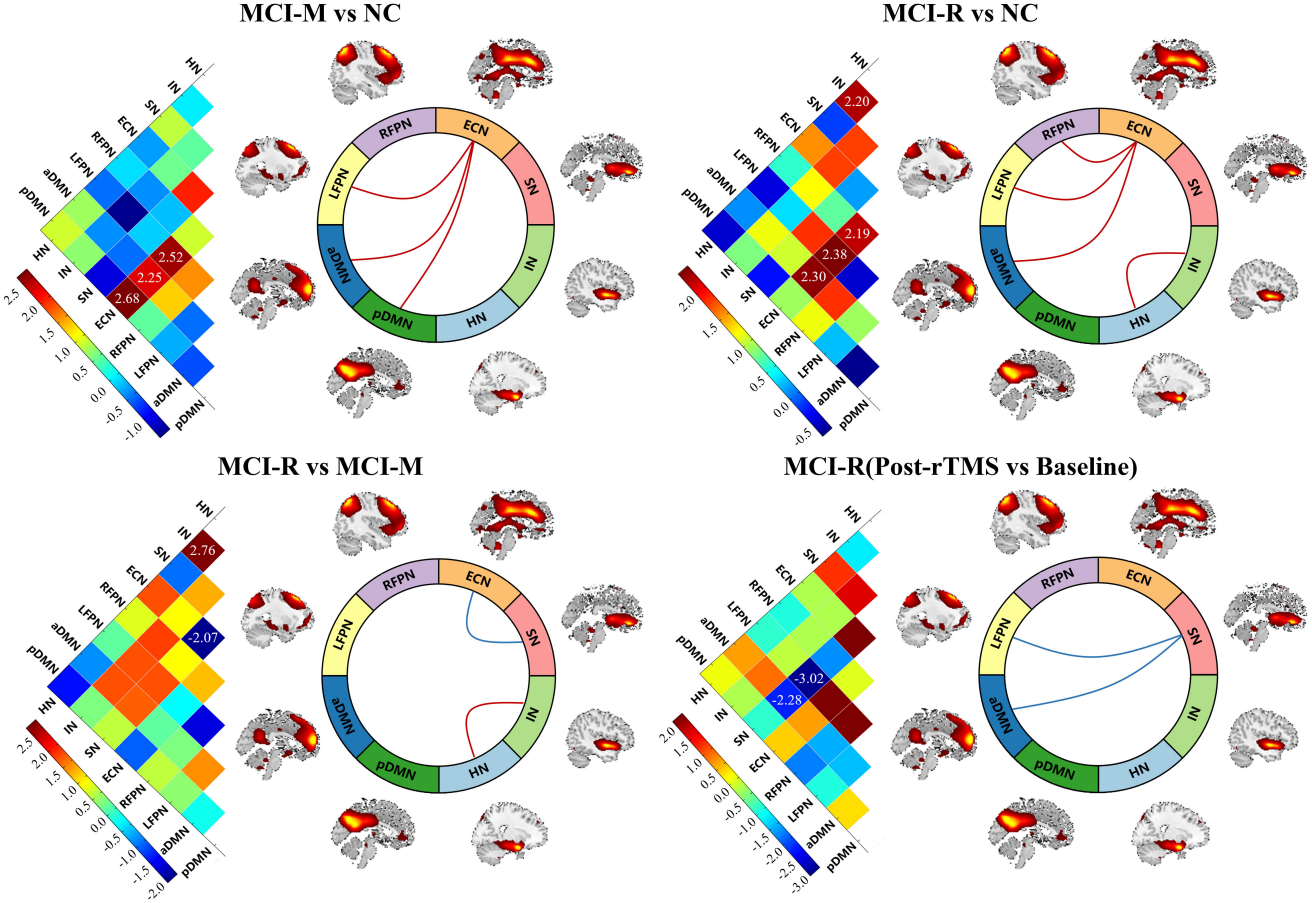
Baseline inter-functional connectivity of the RSNs showed significant difference between MCI and NC, and between MCI-R and MCI-M. MCI-R also showed significant changes in inter-functional connectivity after rTMS. MCI-M, MCI maintainers; MCI-R, MCI reverters; HN, hippocampal network; IN, insular network; SN, salience network; aDMN, anterior default mode network; pDMN, posterior default mode network; ECN, executive control network; LFPN, left frontoparietal network; RFPN, right frontoparietal parietal network.

Additionally, the comparison of baseline intra-functional connectivity of each RSN between MCI-M and MCI-R was conducted. Significant functional connectivity differences were observed in the right precuneus and angular gyrus in aDMN; right inferior parietal lobe in ECN; and right inferior parietal lobe in RFPN between MCI-M and MCI-R (p < 0.005, alphasim corrected) (**Figure 3**). No significant changes in intra-functional connectivity were found in either the MCI-R or MCI-M group post-rTMS. The brain regions showing significant functional connectivity differences between MCI-M and NC and between MCI-R and MCI-M are listed in **Table 2**.

**Table 2:** Regions showing significant functional connectivity differences between MCI-M and NC and between MCI-R and MCI-M.

RSN	Brain Region	Side	Peak Coordinate	Peak Intensity	Cluster Size (Voxels)
MCI-M vs NC					
**pDMN**	Posterior cingulate cortex	Left	−15, −46, 28	5.25	69
	Precuneus	Left	−9, −58, 31	3.98	38
**ECN**	Inferior Parietal Lobe	Right	39, −46, 55	5.47	55
**LFPN**	Superior Frontal Gyrus	Left	−12, 47, 34	6.45	88
	Middle Frontal Gyrus	Left	−35, 29, 28	4.81	60
	Inferior Parietal Lobe	Left	−45, −43, 40	5.57	50
**RFPN**	Superior Frontal Gyrus	Right	15, 47, 40	5.32	38
	Middle Frontal Gyrus	Right	30, 41, 34	5.13	32
MCI-R vs MCI-M					
**aDMN**	Precuneus	Right	15, −61, 46	−5.23	17
	Angular Gyrus	Right	30, −61, 46	−4.75	11
**ECN**	Inferior Parietal Lobe	Right	39, −46, 55	−4.87	25
**RFPN**	Inferior Parietal Lobe	Right	27, −40, 43	−4.24	38

^*^ RSN, resting state network; pDMN, posterior default mode network; aDMN, anterior default mode network; ECN, executive control network; LFPN, left frontoparietal network; RPFN, right frontoparietal network; MCI-R, MCI reverters; NC, normal control; MRI-M, MCI maintainers.

### Differences of inter-functional connectivity among RSNs between-groups and changes within-group

Similar to the comparison of intra-functional connectivity of each RSN, these comparisons were performed in inter-functional connectivity among RSNs. We observed that the inter-functional connectivity between ECN and LFPN, ECN and aDMN, ECN and pDMN of MCI-M were significantly stronger than NC (p < 0.05); the inter-functional connectivity between ECN and LFPN, ECN and RPFN, ECN and aDMN, and between HN and IN were significantly stronger in MCI-R relative to NC (p < 0.05). Compared to MCI-M, participants of MCI-R showed stronger inter-functional connectivity between HN and IN, and weaker inter-functional connectivity between ECN and SN (p < 0.05). Significant changes in inter-functional connectivity were only detected in MCI-R after rTMS relative to baseline, which showed significantly decreased inter-functional connectivity between SN and LFPN, and between SN and aDMN (p < 0.05).

### Correlation between functional connectivity and cognitive improvement

**Figure 4:**
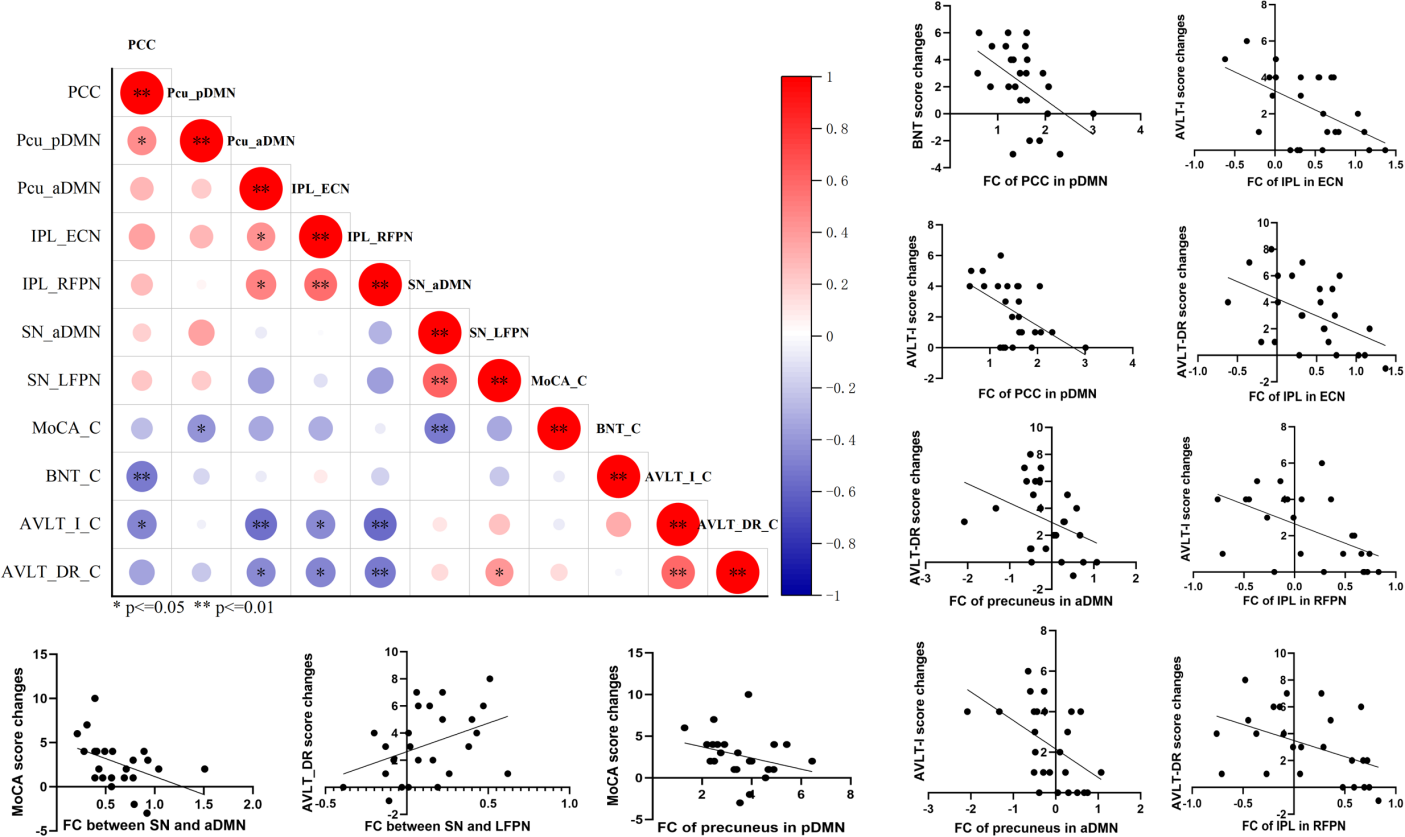
The correlation analysis revealed significant correlation between baseline functional connectivity of RSNs and cognitive improvements in general cognitive function, memory function, and language function. p<=0.05, **p<=0.01.

After completing the comparison of functional connectivity, these brain regions in each RSN and connections between RSNs showing significant differences were considered to be related to the reversion of MCI. Therefore, we examined the relationship between functional connectivity and clinical cognitive improvement after rTMS. First, we computed the correlation coefficient of baseline functional connectivity and cognitive changes of MCI. The results showed that changes in MoCA were significantly associated with the baseline functional connectivity of precuneus in pDMN (r = −0.418, p = 0.038) and inter-functional connectivity between SN and aDMN (r = −0.506, p = 0.010); changes in BNT were significantly associated with the baseline functional connectivity of posterior cingulate cortex in pDMN (r = −0.517, p = 0.008); changes in AVLT-I were significantly associated with the baseline functional connectivity of posterior cingulate cortex in pDMN (r = −0.469, p = 0.018), precuneus in aDMN (r = −0.543, p = 0.005), inferior parietal lobe in ECN (r = −0.453, p = 0.023), and inferior parietal lobe in RFPN (r = −0.571, p = 0.003); changes in AVLT-DR were significantly associated with the baseline functional connectivity of precuneus in aDMN (r = −0.445, p = 0.026), inferior parietal lobe in ECN (r = −0.472, p = 0.017), inferior parietal lobe in RFPN (r = −0.512, p = 0.009), and inter-functional connectivity between SN and aDMN (r = 0.417, p = 0.038) (**Figure 4**). These indicate that the more severe the damage to baseline functional connectivity, the less effect of rTMS on cognitive function in MCI.

**Figure 5:**
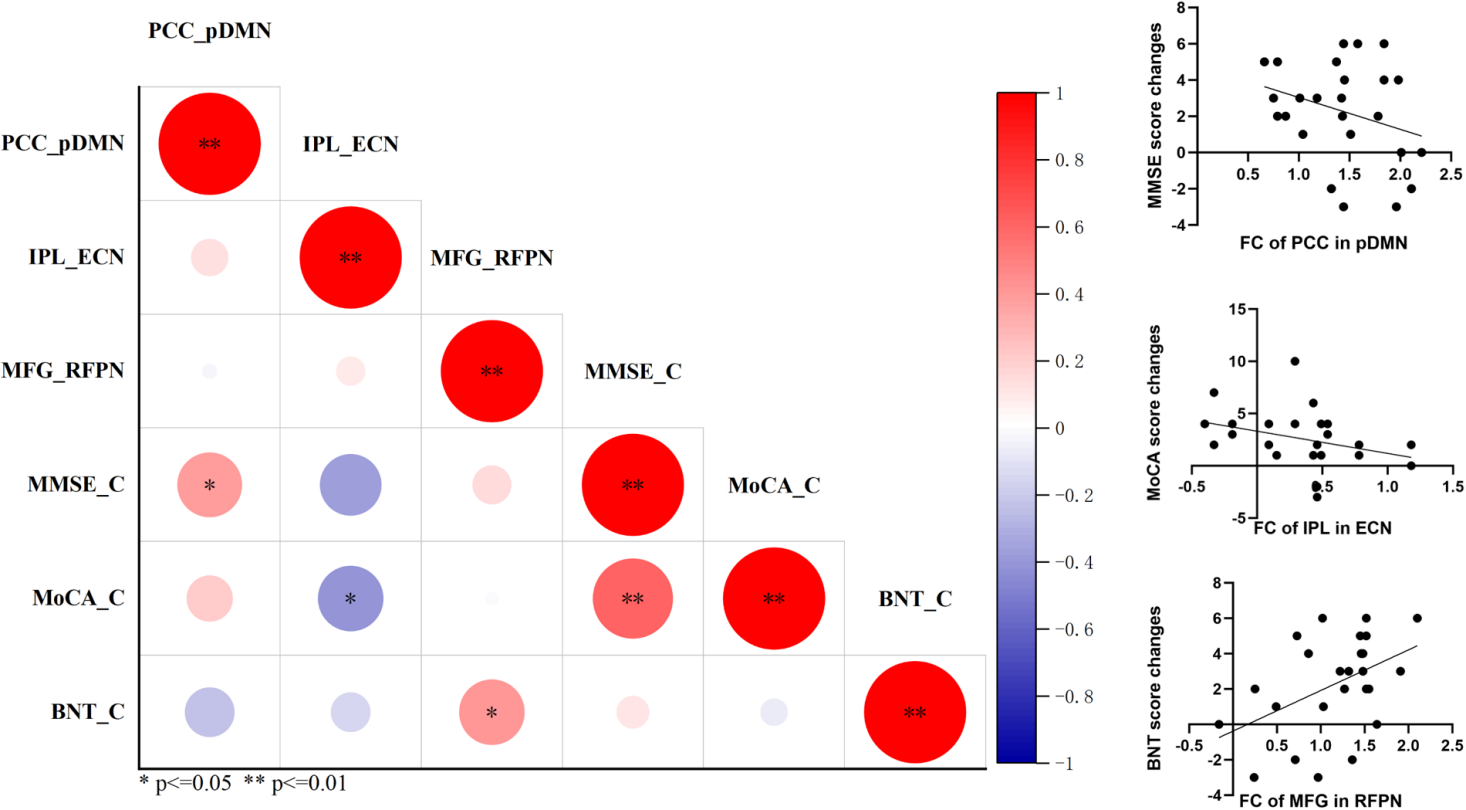
The correlation analysis revealed significant correlation between functional connectivity of RSNs at follow-up and cognitive improvements in general cognitive function and language function. *p<=0.05, **p<=0.01.

The improvement of cognitive performance after rTMS may be related to the state of brain function at follow-up. Then we calculated the correlation coefficient of functional connectivity at follow-up and cognitive changes in MCI. Significant correlation was also detected between the changes of MMSE after rTMS and functional connectivity of posterior cingulate cortex in pDMN at follow-up (r = 0.400, p = 0.048); between the changes of MoCA and functional connectivity of inferior parietal lobe in ECN (r = −0.414 p = 0.040); and between the changes of BNT and inter-functional connectivity of middle frontal gyrus in RFPN (r = 0.409 p = 0.042) (**Figure 5**). This suggests that the improvement of cognitive function in MCI after rTMS is related to the restoration of functional connectivity.

### Possible factors associated with the reversion of MCI after rTMS

**Figure 6:**
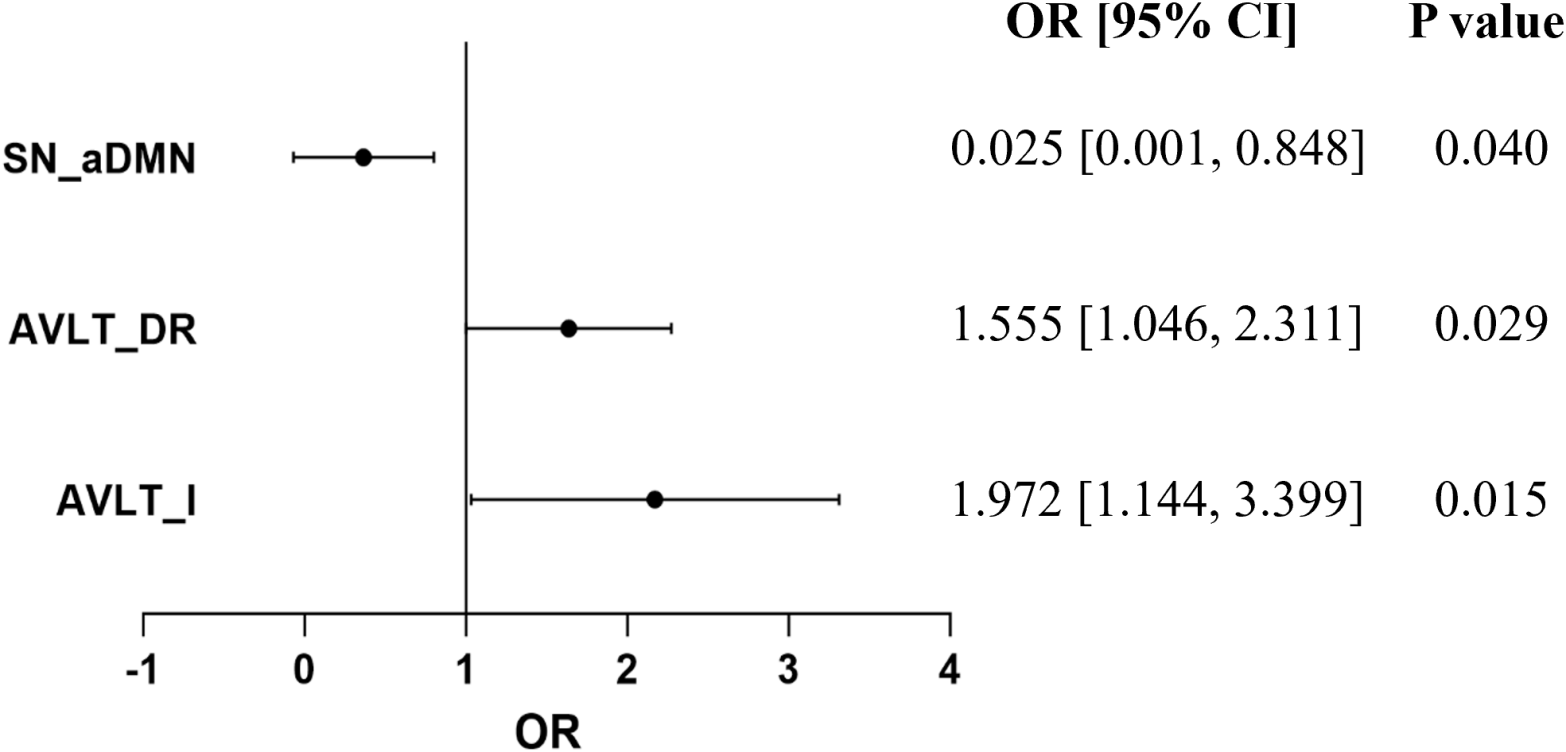
Logistic regression analysis revealed that the score changes of AVLT-I, AVLT-DR after rTMS therapy, and baseline inter-functional connectivity between SN and aDMN may be the possible factors related to the reversion of MCI.

To further explore the possible factors closely associated with the reversion of MCI patients after rTMS, binary logistic regression analysis was performed. The results revealed that the possibility of the reversion of MCI to normal was significantly associated with changes in AVLT-I, AVLT-DR, and inter-functional connectivity between SN and aDMN (odds ratio = 1.972, 1.555, and 0.025; p = 0.015, 0.029, and 0.040) (**Figure 6**).

### Predictors of reversion of MCI patients after rTMS

**Figure 7:**
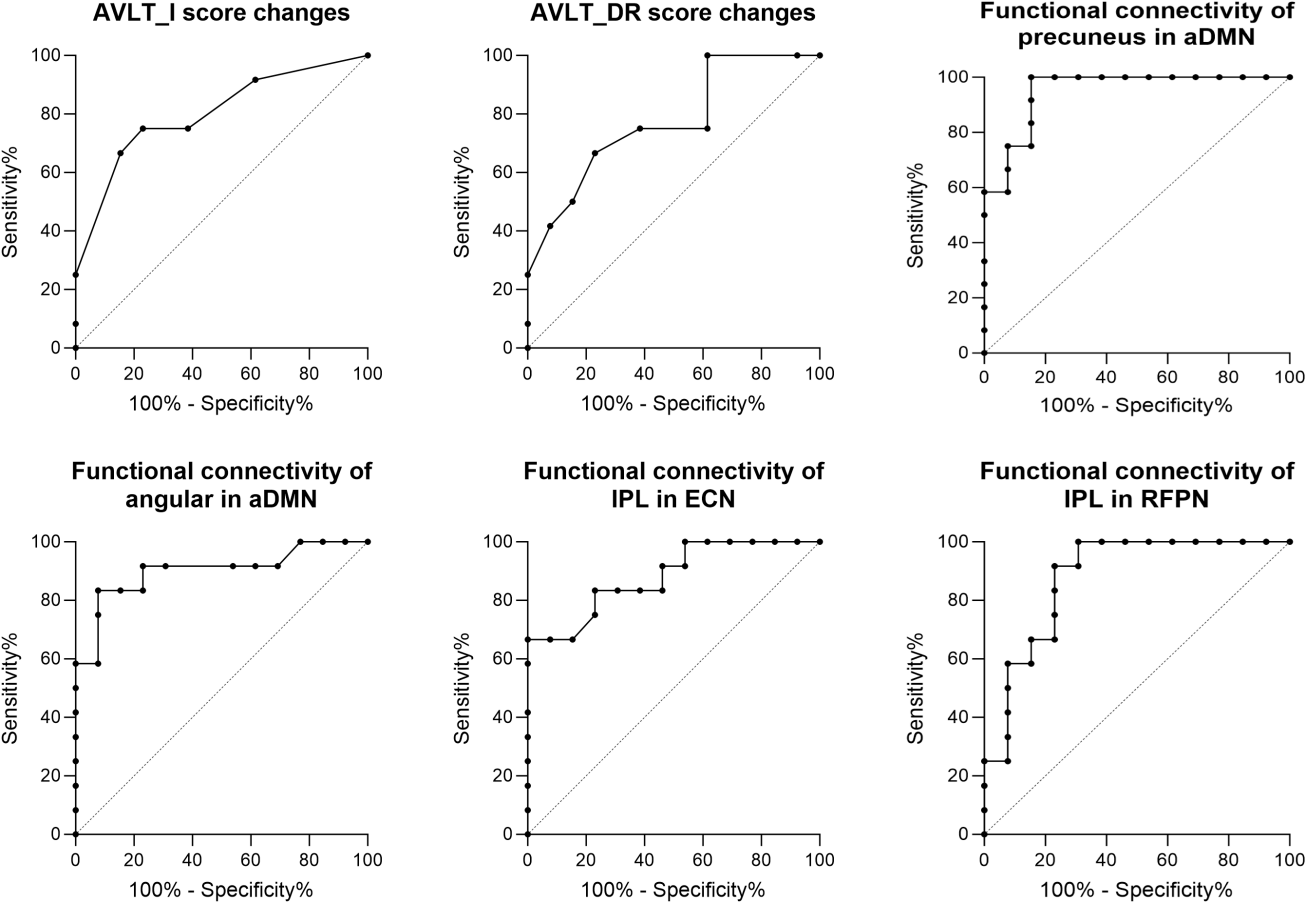
ROC analysis showed predictive value of the score changes of AVLT-I and AVLT-DR after rTMS therapy, and baseline functional connectivity of DMN, ECN, and RFPN for the reversion of MCI after rTMS.

**Figure 7** shows the results of the classification between MCI-M and MCI-R based on changes in cognitive performance and baseline functional connectivity. These indicators were found to be significantly different between MCI-R and MCI-M in previous comparison analysis. ROC curves were used to characterize the predictive value of these indicators. The area under the curve (AUC) of the change in AVLT-I after rTMS was 0.80 (p = 0.01) with a sensitivity of 75.00% and a specificity of 76.92%; the AUC of the change in AVLT-DR after rTMS was 0.77 (p = 0.02) with a sensitivity of 66.67% and a specificity of 76.92%; the AUC of the baseline functional connectivity of precuneus in aDMN was 0.95 (p < 0.001) with a sensitivity of 84.62% and a specificity of 100.00%; the AUC of the baseline functional connectivity of angular in aDMN was 0.90 (p < 0.001) with a sensitivity of 92.33% and a specificity of 83.33%; the AUC of the baseline functional connectivity of inferior parietal lobe in ECN was 0.88 (p < 0.001) with a sensitivity of 100.00% and a specificity of 66.67%; and the AUC of the baseline functional connectivity of inferior parietal lobe in RFPN was 0.88(p < 0.001) with a sensitivity of 69.23% and a specificity of 100.00%. These results suggest that the efficacy of rTMS in immediate and delayed recall memory function could predict the reversion of MCI to normal. In fact, before rTMS therapy, the baseline functional connectivity of DMN, ECN, and RFPN can predict the reversion and demonstrate a superior predictive value.

## Discussion

The present study delved into the behavioral and physiological distinctions between MCI-R and MCI-M, and assessed the impact of rTMS on the reversion of MCI to normal cognitive function to explore the possible predictive biomarkers. Our findings indicate that MCI patients who did not revert to a normal cognitive state at follow-up exhibited more severe cognitive impairments, greater functional connectivity damage, and a less pronounced effect of rTMS. Notably, the superior effect on immediate and delayed recall memory function in MCI-R was significantly associated with the functional connectivity at baseline and follow-up, and demonstrated a strong predictive value for the reversion of MCI. These results suggest that the efficacy of rTMS could promote the reversion of MCI to normal cognition.

### Neuropsychological and functional connectivity differences between MCI-R and MCI-M

Identifying characteristics of individuals with MCI who are likely to revert to normal cognition or remain stable is crucial for clinical trials on early intervention for dementia. Prior research has indicated that fewer APOE e4 alleles, better cognitive function, lower scores of CDR and FAQ scores, larger hippocampal volumes, and lower diastolic blood pressure increase the likelihood of MCI reverting to normal cognition (37, 38). However, this rate is lower in patients with amnestic MCI and multidomain MCI compared to non-amnestic MCI and single domain MCI (37, 39). Additionally, maintaining a healthy lifestyle, particularly engaging in multidomain lifestyle activities (physical, cognitive, and social activities) is beneficial for the reversion of MCI patients to a normal state (40). Our study found more severe cognitive impairments across more domains in MCI-M compared to MCI-R, aligning with previous studies. Particularly, we observed more severe impairments in language function, including naming and verbal fluency in MCI-M. Similar results were reported in only one study, in which the regression analysis observed significant association of delayed recall memory, verbal fluency and BNT with MCI reversion (41). Moreover, another study on PD reported that baseline language function may be associated with progression MCI or PD dementia (42). These findings underscore the importance of healthy language function in the progression and reversion of MCI to a normal state. The severity and extent of cognitive impairments in MCI-M may indicate that the more severe the cognitive impairment in MCI patients, the less likely they are to revert to normal cognition.

Beyond the significant differences in baseline neurocognitive performance, we also observed more severe intra- and inter-functional connectivity damage of RSNs in MCI-M. MCI-M exhibited stronger abnormal functional connectivity in the DMN, ECN, and RFPN, and weaker inter-functional connectivity between HN and IN. Currently, we have not seen any report regarding the difference of functional connectivity or brain activity between MCI-R and MCI-M. A study focusing specifically on MCI-R reported significantly increased intrinsic brain activity including the amplitude of low-frequency of fluctuation, regional homogeneity, and degree centrality in MCI-R compared with healthy controls (43). Another study reported the distinct changes in regional homogeneity of individuals in MCI-R and MCI-M compared to normal controls (44), in which the individuals in MCI-R and MCI-M showed significant changes in different brain regions. The results of these two studies are also completely inconsistent. However, no study has reported the distinction of either brain activity or functional connectivity between MCI-R and MCI-M. In our study, the trends of the functional connectivity changes in both MCI-R and MCI-M groups showed an abnormal increase, with more pronounced changes in MCI-M, indicating more severe impairment. Moreover, the abnormal changes in MCI-M group were more pronounced, which indicated more severe impairment. These performances should be reasonable. These findings are consistent with previous meta-analyses (19, 20) that confirmed the involvement of these brain networks and regions involved in MCI-related cognitive impairments. The DMN, ECN, and FPN are likely the most valuable targets for evaluating the efficiency of interventions for cognitive decline (28). Therefore, better cognitive performance and baseline functional connectivity of DMN, ECN, FPN, and inter-functional connectivity between HN and IN may be associated with the reversion of MCI to normal cognition.

### Effects of rTMS on the reversion of MCI to normal cognition

In addition to the baseline cognitive performance differences between MCI-R and MCI-M, we observed that MCI-R patients experienced more significant improvements in immediate and delayed recall memory function compared to MCI-M. This superior efficacy on immediate recall memory in MCI-R was also significant at follow-up. These results suggest that the significant efficacy of rTMS, especially its notable effects on memory function, may be beneficial for the reversal of MCI to normal. Furthermore, MCI-R patients had relatively milder cognitive impairments than MCI-M at baseline, suggesting that earlier rTMS intervention for MCI could lead to better cognitive improvement, and easier reversion to normal. Previous studies have reported that better cognitive improvement following rTMS treatment is associated with less cognitive impairment (7, 45), slower cognitive decline (7), and higher education level (45). Additionally, more marked cognitive benefits from rTMS intervention have been reported in the early stages of AD (46). Therefore, it is reasonable to expect that MCI patients who experience greater cognitive improvement after rTMS treatment are more likely to return to normal functioning.

Accompanied by cognitive improvement, significant enhancement of intra-functional connectivity between the SN and aDMN, as well as between the SN and LFPN were also observed in MCI-R patients after rTMS treatment. No significant network changes were observed in MCI-M patients after rTMS, suggesting that rTMS did not achieve effective modulation on the neural network in these patients. As we all known that, MCI-related cognitive abnormalities are associated with structural and functional disruptions of brain networks, particularly the high-order triple functioning-related networks of DMN, SN, and ECN (47–51, 20). Aberrant brain activity and functional connectivity within the DMN (15, 16, 52), SN (25, 26), ECN (53), and FPN (17, 26) could be regulated by rTMS intervention. Previous studies have reported significant local activity and functional connectivity changes in these networks after both single-site and dual-targeted rTMS intervention (52), as well as their association with clinical cognitive improvement. Furthermore, it has been proved that functional connectivity changes within DMN could represent a valuable imaging markers of treatment response to predict the after effects of rTMS (15–17). Therefore, the significantly increased functional connectivity observed in MCI-R in our study should be reasonable and could explain the superior effects of rTMS in MCI-R patients after rTMS. Besides, the changes in functional connectivity suggest that rTMS effectively modulates the networks in MCI-R patients, while it did not exert regulatory effects in MCI-M patients. This may be the possible reason why MCI-M was unable to revert to normal.

### Imaging and behavioral factors associated with the reversion of MCI after rTMS

To explore the possible factors involving baseline functional connectivity, effects of rTMS, and basic information of MCI patients, we utilized correlation analysis, logistic regression, and ROC analysis. Consistent with the behavioral and imaging comparison results, significant correlations were mainly observed between the changes in memory function after rTMS treatment and the baseline functional connectivity of DMN, ECN, SN, and FPN. Furthermore, the changes in immediate and delayed recall memory function following rTMS, as well as the functional connectivity of the aforementioned networks exhibited excellent predictive value for MCI-M. These results further indicate that the effects of rTMS on memory function is conductive to reversing MCI to normal. The state of MCI after rTMS intervention can be predicted by using functional connectivity of high-order cognition-related brain networks and the effects of rTMS. Previous studies have proved the predictive value of functional connectivity changes in DMN to the after effects of rTMS (15–17). However, there have been no reports to date on indicators that can predict the outcomes of MCI after rTMS or any other intervention. As is well known, the clinical manifestation of MCI is a decline in memory function. Furthermore, numerous studies have demonstrated that rTMS has a significant positive effect on memory improvement (6, 54, 55). Therefore, the enhancement of memory function is likely to play a very important role in the reversal of MCI.

## Limitations

There are several limitations should be considered regarding to our findings. First, the sample size of the study was small. Although, fifty-three MCI participants completed the 10-day rTMS treatment, only about half of the participants underwent an 18-month follow-up after treatment. However, most of the participants who were unable to attend the follow-up due to being in different locations, refusal to participate in the follow-up, inability to establish contact, or death. The treatment effects and positive experience of the rTMS treatment were affirmed by the majority of these individuals. Second, this study lacked of a control group. We had planned to follow up with MCI participants who did not receive rTMS treatment for control comparison. However, due to the sample size and the fact that the majority were unwilling to participate in the follow-up, we only completed the follow-up with a few participants. Further studies with larger sample sizes and control groups are needed to validate these findings.

## Conclusion

In summary, this study demonstrated that the efficacy of rTMS therapy may promote the reversion of MCI to normal cognitive function. Improvement of memory function after rTMS and baseline functional connectivity in DMN, ECN, and FPN can predict the reversion. Moreover, the more severe the cognitive impairment and functional connectivity damage, the less likely MCI is to revert to normal. This underscores the importance of early rTMS intervention for the early prevention of dementia.

## Data Availability

The raw data supporting the conclusions of this article will be made available by the authors, without undue reservation.
